# Supplementary material for: SNPs in Genes Related to DNA Damage Repair in Mycobacterium Tuberculosis: Their Association with Type 2 Diabetes Mellitus and Drug Resistance
Source: Genes (Basel). 2022 Mar 29;13(4):609. doi: 10.3390/genes13040609 (PMC9029044; doi:10.3390/genes13040609)
Supplement: Supplementary file 1 [file genes-13-00609-s001.zip › genes-1610551-supplementary.pdf]

SUPPLEMENTARY MATERIAL

Supplementary Table S1. Data of the analyzed sequences

ID	Country	T2DM	Sensitivity profile	Publication status
SRR5153073	Azerbaijan	Yes	Sensitive	Published
SRR6356936	Azerbaijan	Yes	Drug resistant	Published
SRR6356938	Azerbaijan	Yes	Drug resistant	Published
SRR6356975	Azerbaijan	Yes	Sensitive	Published
SRR6357008	Azerbaijan	Yes	Drug resistant	Published
SRR13232428	Belarus	No	Drug resistant	Published
SRR13232467	Belarus	Yes	Drug resistant	Published
SRR13232498	Belarus	No	Drug resistant	Published
SRR13232543	Belarus	No	Drug resistant	Published
SRR13232554	Belarus	No	Drug resistant	Published
SRR13232573	Belarus	Yes	Drug resistant	Published
SRR13232580	Belarus	No	Drug resistant	Published
SRR13232591	Belarus	Yes	Drug resistant	Published
SRR13232681	Belarus	No	Drug resistant	Published
SRR13232706	Belarus	No	Drug resistant	Published
SRR13232756	Belarus	No	Drug resistant	Published
SRR13232779	Belarus	Yes	Drug resistant	Published
SRR6458398	Belarus	No	Sensitive	Published
SRR6458409	Belarus	Yes	Sensitive	Published
SRR6458433	Belarus	No	Drug resistant	Published
SRR6458436	Belarus	No	Drug resistant	Published
SRR6458461	Belarus	No	Sensitive	Published
SRR6458462	Belarus	No	Drug resistant	Published
SRR6458464	Belarus	No	Sensitive	Published
SRR10397092	Georgia	No	Drug resistant	Published
SRR10397107	Georgia	Yes	Drug resistant	Published
SRR10397108	Georgia	No	Drug resistant	Published
SRR10397114	Georgia	No	Drug resistant	Published
SRR10397170	Georgia	Yes	Drug resistant	Published
SRR10397186	Georgia	No	Drug resistant	Published
SRR10397193	Georgia	No	Drug resistant	Published
SRR10397202	Georgia	No	Sensitive	Published
SRR10397218	Georgia	Yes	Drug resistant	Published
SRR10397250	Georgia	No	Sensitive	Published
SRR10397253	Georgia	No	Drug resistant	Published
SRR10397258	Georgia	No	Sensitive	Published
SRR10397266	Georgia	No	Drug resistant	Published

SRR10397269	Georgia	No	Sensitive	Published
SRR11033589	Georgia	No	Sensitive	Published
SRR11033590	Georgia	No	Sensitive	Published
SRR11033593	Georgia	No	Drug resistant	Published
SRR11033594	Georgia	No	Sensitive	Published
SRR11033595	Georgia	No	Sensitive	Published
SRR11033596	Georgia	No	Sensitive	Published
SRR11033597	Georgia	No	Sensitive	Published
SRR11033600	Georgia	No	Drug resistant	Published
SRR11033601	Georgia	No	Sensitive	Published
SRR11033602	Georgia	No	Sensitive	Published
SRR11033604	Georgia	No	Sensitive	Published
SRR11033605	Georgia	No	Sensitive	Published
SRR11033606	Georgia	No	Sensitive	Published
SRR11033607	Georgia	No	Sensitive	Published
SRR11033608	Georgia	No	Sensitive	Published
SRR11033609	Georgia	No	Sensitive	Published
SRR11033611	Georgia	No	Drug resistant	Published
SRR11033612	Georgia	No	Sensitive	Published
SRR11033613	Georgia	No	Sensitive	Published
SRR11033615	Georgia	No	Sensitive	Published
SRR11033617	Georgia	No	Sensitive	Published
SRR11033618	Georgia	No	Sensitive	Published
SRR11033619	Georgia	No	Sensitive	Published
SRR11033620	Georgia	No	Drug resistant	Published
SRR11033622	Georgia	No	Sensitive	Published
SRR11033623	Georgia	No	Sensitive	Published
SRR11033624	Georgia	No	Sensitive	Published
SRR11033625	Georgia	No	Drug resistant	Published
SRR11033626	Georgia	No	Sensitive	Published
SRR11033627	Georgia	No	Sensitive	Published
SRR11033628	Georgia	No	Sensitive	Published
SRR11033631	Georgia	No	Sensitive	Published
SRR11033633	Georgia	No	Sensitive	Published
SRR11033634	Georgia	No	Drug resistant	Published
SRR11033635	Georgia	No	Sensitive	Published
SRR11033637	Georgia	No	Drug resistant	Published
SRR11033638	Georgia	No	Sensitive	Published
SRR11033642	Georgia	No	Sensitive	Published
SRR11033646	Georgia	No	Drug resistant	Published
SRR11033647	Georgia	No	Sensitive	Published

SRR11033648	Georgia	No	Sensitive	Published
SRR11033649	Georgia	No	Drug resistant	Published
SRR11033650	Georgia	No	Sensitive	Published
SRR11033652	Georgia	No	Sensitive	Published
SRR11033653	Georgia	No	Sensitive	Published
SRR11033656	Georgia	No	Sensitive	Published
SRR11033657	Georgia	No	Sensitive	Published
SRR11033658	Georgia	No	Drug resistant	Published
SRR11033659	Georgia	No	Sensitive	Published
SRR11033660	Georgia	No	Sensitive	Published
SRR11033666	Georgia	No	Sensitive	Published
SRR11033667	Georgia	No	Sensitive	Published
SRR11033669	Georgia	No	Sensitive	Published
SRR11033670	Georgia	No	Sensitive	Published
SRR11033672	Georgia	No	Sensitive	Published
SRR11033681	Georgia	No	Drug resistant	Published
SRR11033682	Georgia	No	Sensitive	Published
SRR11033683	Georgia	No	Sensitive	Published
SRR11033684	Georgia	No	Drug resistant	Published
SRR11033687	Georgia	No	Sensitive	Published
SRR11033689	Georgia	No	Sensitive	Published
SRR11033697	Georgia	No	Sensitive	Published
SRR11033700	Georgia	No	Drug resistant	Published
SRR11033702	Georgia	No	Drug resistant	Published
SRR11033707	Georgia	No	Sensitive	Published
SRR11033708	Georgia	No	Sensitive	Published
SRR11033709	Georgia	No	Sensitive	Published
SRR11033712	Georgia	No	Drug resistant	Published
SRR11033714	Georgia	No	Drug resistant	Published
SRR11033720	Georgia	No	Sensitive	Published
SRR11033723	Georgia	No	Sensitive	Published
SRR11033725	Georgia	No	Sensitive	Published
SRR11033733	Georgia	No	Sensitive	Published
SRR11033749	Georgia	No	Sensitive	Published
SRR11033754	Georgia	No	Sensitive	Published
SRR11033757	Georgia	No	Drug resistant	Published
SRR11033762	Georgia	No	Drug resistant	Published
SRR11033763	Georgia	No	Sensitive	Published
SRR11033764	Georgia	No	Sensitive	Published
SRR11033765	Georgia	No	Sensitive	Published
SRR11033766	Georgia	No	Sensitive	Published

SRR11033769	Georgia	No	Sensitive	Published
SRR11033770	Georgia	No	Sensitive	Published
SRR11033771	Georgia	No	Sensitive	Published
SRR11033773	Georgia	No	Sensitive	Published
SRR11033774	Georgia	No	Sensitive	Published
SRR11033776	Georgia	No	Sensitive	Published
SRR11033777	Georgia	No	Sensitive	Published
SRR11033780	Georgia	No	Sensitive	Published
SRR3544717	Georgia	No	Drug resistant	Published
SRR3544722	Georgia	No	Sensitive	Published
SRR3544731	Georgia	No	Drug resistant	Published
SRR3544736	Georgia	No	Sensitive	Published
SRR3544738	Georgia	No	Sensitive	Published
SRR3544742	Georgia	No	Sensitive	Published
SRR3544744	Georgia	No	Sensitive	Published
SRR3544750	Georgia	No	Sensitive	Published
SRR3544752	Georgia	No	Sensitive	Published
SRR5152915	Georgia	No	Sensitive	Published
SRR5152927	Georgia	No	Drug resistant	Published
SRR5152929	Georgia	No	Sensitive	Published
SRR5152938	Georgia	No	Sensitive	Published
SRR5153088	Georgia	Yes	Drug resistant	Published
SRR5153089	Georgia	No	Drug resistant	Published
SRR5153095	Georgia	No	Drug resistant	Published
SRR5153213	Georgia	No	Sensitive	Published
SRR5153216	Georgia	No	Drug resistant	Published
SRR5153240	Georgia	No	Drug resistant	Published
SRR5153266	Georgia	No	Sensitive	Published
SRR5153309	Georgia	No	Drug resistant	Published
SRR5153316	Georgia	No	Drug resistant	Published
SRR5153332	Georgia	Yes	Drug resistant	Published
SRR7516353	Georgia	Yes	Drug resistant	Published
SRR7516357	Georgia	No	Sensitive	Published
SRR7516400	Georgia	No	Drug resistant	Published
SRR7516407	Georgia	No	Drug resistant	Published
SRR7516420	Georgia	No	Sensitive	Published
SRR7516434	Georgia	No	Drug resistant	Published
SRR7516437	Georgia	Yes	Drug resistant	Published
SRR7516445	Georgia	No	Sensitive	Published
SRR10808355	Indonesia	Yes	Sensitive	Published
SRR10808388	Indonesia	Yes	Sensitive	Published

SRR10808432	Indonesia	Yes	Sensitive	Published
SRR10808450	Indonesia	Yes	Sensitive	Published
SRR10808546	Indonesia	Yes	Drug resistant	Published
SRR10808568	Indonesia	Yes	Sensitive	Published
SRR10808708	Indonesia	Yes	Sensitive	Published
SRR10808712	Indonesia	Yes	Sensitive	Published
SRR10808713	Indonesia	Yes	Sensitive	Published
SRR10808722	Indonesia	Yes	Sensitive	Published
SRR10808725	Indonesia	Yes	Sensitive	Published
SRR10808739	Indonesia	Yes	Sensitive	Published
SRR10808758	Indonesia	Yes	Sensitive	Published
SRR10808770	Indonesia	Yes	Drug resistant	Published
SRR10808811	Indonesia	Yes	Sensitive	Published
SRR10808934	Indonesia	Yes	Sensitive	Published
SRR10808965	Indonesia	Yes	Sensitive	Published
SRR10808969	Indonesia	Yes	Sensitive	Published
SRR10808970	Indonesia	Yes	Drug resistant	Published
SRR10808990	Indonesia	Yes	Sensitive	Published
SRR10808996	Indonesia	Yes	Drug resistant	Published
SRR10809020	Indonesia	Yes	Sensitive	Published
SRR10809030	Indonesia	Yes	Sensitive	Published
SRR10809047	Indonesia	Yes	Sensitive	Published
SRR10809049	Indonesia	Yes	Sensitive	Published
SRR10809050	Indonesia	Yes	Sensitive	Published
SRR10809054	Indonesia	Yes	Sensitive	Published
SRR10809068	Indonesia	Yes	Sensitive	Published
SRR10809070	Indonesia	Yes	Sensitive	Published
SRR10809072	Indonesia	Yes	Sensitive	Published
SRR10809083	Indonesia	Yes	Sensitive	Published
SRR10809084	Indonesia	Yes	Sensitive	Published
SRR10809085	Indonesia	Yes	Sensitive	Published
SRR10809086	Indonesia	Yes	Drug resistant	Published
SRR10809089	Indonesia	Yes	Sensitive	Published
SRR10809103	Indonesia	Yes	Drug resistant	Published
SRR10809104	Indonesia	Yes	Sensitive	Published
SRR10809113	Indonesia	Yes	Sensitive	Published
SRR10809116	Indonesia	Yes	Sensitive	Published
SRR10809123	Indonesia	Yes	Drug resistant	Published
SRR10809136	Indonesia	Yes	Sensitive	Published
SRR10809137	Indonesia	Yes	Sensitive	Published
SRR10809140	Indonesia	Yes	Drug resistant	Published

SRR10809144	Indonesia	Yes	Sensitive	Published
SRR10809160	Indonesia	Yes	Sensitive	Published
SRR10809171	Indonesia	Yes	Sensitive	Published
SRR10809189	Indonesia	Yes	Sensitive	Published
SRR10809194	Indonesia	Yes	Sensitive	Published
SRR10809199	Indonesia	Yes	Sensitive	Published
SRR10809207	Indonesia	Yes	Drug resistant	Published
SRR10809213	Indonesia	Yes	Sensitive	Published
SRR13232424	Kazakhstan	No	Drug resistant	Published
SRR13232487	Kazakhstan	No	Sensitive	Published
SRR13232622	Kazakhstan	Yes	Sensitive	Published
ERR3148156	Mexico	No	Drug resistant	Published
ERR3148157	Mexico	Yes	Sensitive	Published
ERR3148163	Mexico	Yes	Drug resistant	Published
ERR3148165	Mexico	Yes	Drug resistant	Published
ERR3148170	Mexico	Yes	Drug resistant	Published
ERR3148171	Mexico	Yes	Drug resistant	Published
ERR3148172	Mexico	Yes	Drug resistant	Published
ERR3148173	Mexico	Yes	Drug resistant	Published
ERR3148174	Mexico	Yes	Drug resistant	Published
ERR3148175	Mexico	Yes	Drug resistant	Published
ERR3148176	Mexico	Yes	Drug resistant	Published
ERR3148184	Mexico	No	Drug resistant	Published
ERR3148186	Mexico	Yes	Drug resistant	Published
ERR3148187	Mexico	Yes	Drug resistant	Published
ERR3148188	Mexico	Yes	Drug resistant	Published
ERR3148189	Mexico	Yes	Drug resistant	Published
ERR3148190	Mexico	Yes	Drug resistant	Published
ERR3148191	Mexico	Yes	Drug resistant	Published
ERR3148192	Mexico	Yes	Drug resistant	Published
ERR3148193	Mexico	No	Sensitive	Published
ERR3148195	Mexico	Yes	Sensitive	Published
ERR3148196	Mexico	Yes	Sensitive	Published
ERR3148197	Mexico	Yes	Drug resistant	Published
ERR3148198	Mexico	No	Drug resistant	Published
ERR3148199	Mexico	No	Drug resistant	Published
ERR3148200	Mexico	Yes	Drug resistant	Published
ERR3148201	Mexico	No	Drug resistant	Published
ERR3148202	Mexico	No	Drug resistant	Published
ERR3148203	Mexico	No	Drug resistant	Published
ERR3148204	Mexico	No	Drug resistant	Published

ERR3148205	Mexico	No	Drug resistant	Published
ERR3148207	Mexico	Yes	Sensitive	Published
ERR3148208	Mexico	Yes	Drug resistant	Published
ERR3148209	Mexico	Yes	Sensitive	Published
ERR3148210	Mexico	No	Sensitive	Published
ERR3148211	Mexico	Yes	Drug resistant	Published
ERR3148212	Mexico	No	Sensitive	Published
ERR3148213	Mexico	No	Drug resistant	Published
ERR3148216	Mexico	Yes	Drug resistant	Published
ERR3148217	Mexico	Yes	Drug resistant	Published
ERR3148218	Mexico	No	Sensitive	Published
ERR3148220	Mexico	No	Sensitive	Published
ERR3148222	Mexico	Yes	Drug resistant	Published
ERR3148224	Mexico	No	Sensitive	Published
ERR3148225	Mexico	No	Drug resistant	Published
ERR3148226	Mexico	Yes	Sensitive	Published
ERR3148229	Mexico	No	Sensitive	Published
SRR10379888	Moldova	No	Sensitive	Published
SRR10379892	Moldova	No	Drug resistant	Published
SRR10379896	Moldova	No	Drug resistant	Published
SRR10379899	Moldova	No	Drug resistant	Published
SRR10379901	Moldova	No	Sensitive	Published
SRR10379903	Moldova	No	Drug resistant	Published
SRR10379906	Moldova	No	Drug resistant	Published
SRR10379908	Moldova	No	Drug resistant	Published
SRR10379912	Moldova	No	Drug resistant	Published
SRR10379915	Moldova	No	Sensitive	Published
SRR10379917	Moldova	No	Drug resistant	Published
SRR10379932	Moldova	No	Drug resistant	Published
SRR10379936	Moldova	No	Drug resistant	Published
SRR10379938	Moldova	No	Sensitive	Published
SRR10379944	Moldova	No	Drug resistant	Published
SRR10379945	Moldova	Yes	Drug resistant	Published
SRR10379971	Moldova	No	Drug resistant	Published
SRR10379987	Moldova	No	Drug resistant	Published
SRR10379988	Moldova	No	Sensitive	Published
SRR10379990	Moldova	Yes	Drug resistant	Published
SRR10380002	Moldova	No	Drug resistant	Published
SRR10380006	Moldova	No	Sensitive	Published
SRR10380009	Moldova	No	Drug resistant	Published
SRR10380011	Moldova	No	Drug resistant	Published

SRR10380014	Moldova	No	Drug resistant	Published
SRR10380016	Moldova	No	Sensitive	Published
SRR10380027	Moldova	No	Sensitive	Published
SRR10380037	Moldova	No	Drug resistant	Published
SRR10380039	Moldova	No	Drug resistant	Published
SRR10380043	Moldova	No	Drug resistant	Published
SRR10380047	Moldova	Yes	Drug resistant	Published
SRR10380057	Moldova	Yes	Drug resistant	Published
SRR10380076	Moldova	Yes	Drug resistant	Published
SRR10380130	Moldova	No	Drug resistant	Published
SRR10380154	Moldova	No	Sensitive	Published
SRR10380160	Moldova	No	Drug resistant	Published
SRR10380163	Moldova	No	Sensitive	Published
SRR10380175	Moldova	Yes	Sensitive	Published
SRR10380179	Moldova	No	Sensitive	Published
SRR10380193	Moldova	No	Sensitive	Published
SRR10380195	Moldova	No	Sensitive	Published
SRR10380211	Moldova	Yes	Sensitive	Published
SRR10380212	Moldova	No	Sensitive	Published
SRR10380213	Moldova	No	Drug resistant	Published
SRR10380215	Moldova	No	Drug resistant	Published
SRR10380216	Moldova	No	Drug resistant	Published
SRR10380217	Moldova	No	Drug resistant	Published
SRR10380219	Moldova	No	Sensitive	Published
SRR10380221	Moldova	No	Sensitive	Published
SRR10380232	Moldova	No	Sensitive	Published
SRR10380235	Moldova	No	Sensitive	Published
SRR10380236	Moldova	No	Sensitive	Published
SRR10380240	Moldova	No	Drug resistant	Published
SRR3743493	Moldova	Yes	Sensitive	Published
SRR5153902	Moldova	Yes	Drug resistant	Published
SRR10808334	Peru	Yes	Sensitive	Published
SRR10808337	Peru	Yes	Drug resistant	Published
SRR10808342	Peru	Yes	Sensitive	Published
SRR10808344	Peru	Yes	Sensitive	Published
SRR10808353	Peru	Yes	Drug resistant	Published
SRR10808370	Peru	Yes	Drug resistant	Published
SRR10808409	Peru	Yes	Drug resistant	Published
SRR10808462	Peru	Yes	Sensitive	Published
SRR10808467	Peru	Yes	Sensitive	Published
SRR10808472	Peru	Yes	Sensitive	Published

SRR10808520	Peru	Yes	Drug resistant	Published
SRR10808536	Peru	Yes	Sensitive	Published
SRR10808543	Peru	Yes	Sensitive	Published
SRR10808572	Peru	Yes	Drug resistant	Published
SRR10808574	Peru	Yes	Drug resistant	Published
SRR10808607	Peru	Yes	Sensitive	Published
SRR10808641	Peru	Yes	Sensitive	Published
SRR10808654	Peru	Yes	Sensitive	Published
SRR10808670	Peru	Yes	Drug resistant	Published
SRR10808688	Peru	Yes	Drug resistant	Published
SRR10808704	Peru	Yes	Drug resistant	Published
SRR10808810	Peru	Yes	Sensitive	Published
SRR10808827	Peru	Yes	Sensitive	Published
SRR10808882	Peru	Yes	Sensitive	Published
SRR10808917	Peru	Yes	Drug resistant	Published
SRR10808924	Peru	Yes	Sensitive	Published
SRR10808926	Peru	Yes	Sensitive	Published
SRR10808928	Peru	Yes	Sensitive	Published
SRR10808948	Peru	Yes	Drug resistant	Published
SRR10525318	Romania	No	Drug resistant	Published
SRR10525325	Romania	No	Drug resistant	Published
SRR10525360	Romania	Yes	Drug resistant	Published
SRR10525371	Romania	No	Drug resistant	Published
SRR3544723	Romania	No	Sensitive	Published
SRR3743199	Romania	Yes	Sensitive	Published
SRR5486869	Romania	Yes	Drug resistant	Published
SRR5486894	Romania	No	Drug resistant	Published
SRR5486901	Romania	No	Drug resistant	Published
SRR7592322	Romania	No	Drug resistant	Published
SRR7592333	Romania	No	Drug resistant	Published
SRR7592379	Romania	No	Drug resistant	Published
SRR9738478	Romania	No	Sensitive	Published
SRR9738483	Romania	No	Drug resistant	Published
SRR9738491	Romania	No	Sensitive	Published
SRR9738494	Romania	No	Drug resistant	Published
SRR9738495	Romania	Yes	Drug resistant	Published
SRR9738514	Romania	No	Drug resistant	Published
SRR9738528	Romania	No	Drug resistant	Published
SRR9738545	Romania	No	Drug resistant	Published
SRR9738552	Romania	No	Drug resistant	Published
G1036	Spain	Yes	Sensitive	not published

G1107	Spain	Yes	Sensitive	not published
G1243	Spain	Yes	Sensitive	not published
G1302m	Spain	Yes	Sensitive	not published
G1303	Spain	Yes	Sensitive	not published
G1304	Spain	Yes	Sensitive	not published
G1312	Spain	Yes	Drug resistant	not published
G1320	Spain	Yes	Sensitive	not published
G1327	Spain	Yes	Sensitive	not published
G1359	Spain	Yes	Sensitive	not published
G1449	Spain	Yes	Sensitive	not published
G1509	Spain	Yes	Sensitive	not published
G1521	Spain	Yes	Drug resistant	not published
G1523	Spain	Yes	Drug resistant	not published
G1532	Spain	Yes	Sensitive	not published
G1533	Spain	Yes	Sensitive	not published
G1568	Spain	Yes	Sensitive	not published
G1573	Spain	Yes	Drug resistant	not published
G1583	Spain	Yes	Sensitive	not published
G1617	Spain	Yes	Sensitive	not published
G1657	Spain	Yes	Drug resistant	not published
G1672	Spain	Yes	Drug resistant	not published
G1684	Spain	Yes	Sensitive	not published
G1728	Spain	Yes	Sensitive	not published
G1731	Spain	Yes	Sensitive	not published
G1763	Spain	Yes	Sensitive	not published
G1764	Spain	Yes	Sensitive	not published
G1765	Spain	Yes	Sensitive	not published
G1766	Spain	Yes	Sensitive	not published
G1782	Spain	Yes	Sensitive	not published
G1792	Spain	Yes	Sensitive	not published
G1910	Spain	Yes	Sensitive	not published
G1934	Spain	Yes	Drug resistant	not published
G271	Spain	Yes	Drug resistant	not published
G357m	Spain	Yes	Sensitive	not published

T2DM: Type 2 diabetes mellitus.

Supplementary Table S2. Genes related to DNA damage repair in *M. Tuberculosis*

Gene name	Identifier	Start	End	Gene length	Orientation	Essentiality
**RecF**	Rv0003	3280	4437	1158	+	nonessential
**SSBa**	Rv0054	58586	59080	495	+	nonessential
**PolD2**	Rv0269c	323338	324531	1194	-	nonessential
**MutT3**	Rv0413	499713	500366	654	+	nonessential
**XthA**	Rv0427c	516017	516892	876	-	essential
**RecD**	Rv0629c	720005	721732	1728	-	nonessential
**RecB**	Rv0630c	721729	725013	3285	-	nonessential
**RecC**	Rv0631c	725013	728306	3294	-	nonessential
**End (Nfo)**	Rv0670	769792	770550	759	+	essential
**Ku**	Rv0937c	1045199	1046020	822	-	nonessential
**LigD**	Rv0938	1046136	1048415	2280	+	nonessential
**Fpg2**	Rv0944	1053765	1054241	477	+	nonessential
**UvrD1**	Rv0949	1058260	1060575	2316	+	essential
**Mfd**	Rv1020	1138967	1142671	3705	+	nonessential
**MazG**	Rv1021	1142671	1143648	978	+	essential
**MutT2**	Rv1160	1286595	1287020	426	+	nonessential
**TagA**	Rv1210	1353522	1354136	615	+	nonessential
**UdgB**	Rv1259	1407339	1408238	900	+	nonessential
**AlkA**	Rv1317c	1477628	1479118	1491	-	nonessential
**NucS**	Rv1321	1484279	1484959	681	+	nonessential
**UvrC**	Rv1420	1594042	1595982	1941	+	essential
**DinB1**	Rv1537	1739856	1741247	1392	+	nonessential
**DnaE1**	Rv1547	1747694	1751248	3555	+	essential
**Ogt / adaB**	Rv1316c	1477134	1477631	498	-	nonessential
**PolA**	Rv1629	1830665	1833379	2715	+	essential
**UvrB**	Rv1633	1837075	1839171	2097	+	nonessential
**UvrA**	Rv1638	1843741	1846659	2919	+	nonessential
**Mpg**	Rv1688	1912979	1913590	612	+	nonessential
**RecN**	Rv1696	1919683	1921446	1764	+	essential
**Rv2119**	Rv2119	2378386	2379222	837	+	nonessential
**Cho**	Rv2191	2453819	2455756	1938	+	nonessential
**RNaseH1**	Rv2228c	2501644	2502738	1095	-	nonessential
**RecO**	Rv2362c	2643461	2644258	798	-	nonessential
**Nei1**	Rv2464c	2766859	2767665	807	-	nonessential
**SSBb**	Rv2478c	2784123	2784608	486	-	nonessential
**RuvX**	Rv2554c	2873258	2873770	513	-	essential
**RuvB**	Rv2592c	2923199	2924233	1035	-	nonessential
**RuvA**	Rv2593c	2924230	2924820	591	-	nonessential
**RuvC**	Rv2594c	2924817	2925383	567	-	nonessential
**RecGwed**	Rv2694c	3011399	3011767	369	-	nonessential
**Dut**	Rv2697c	3013683	3014147	465	-	essential
**RecX**	Rv2736c	3048562	3049086	525	-	nonessential
**RecA**	Rv2737c	3049052	3051424	2373	-	nonessential
**RNaseH2**	Rv2902c	3212162	3212956	795	-	nonessential
**MutM (Fpg)**	Rv2924c	3238601	3239470	870	-	nonessential
**RecG**	Rv2973c	3327733	3329946	2214	-	nonessential

**Ung**	Rv2976c	3332071	3332754	684	-	essential
**MutT1**	Rv2985	3342165	3343118	954	-	nonessential
**LigA**	Rv3014c	3372545	3374620	2073	-	nonessential
**DinB2**	Rv3056	3416705	3417745	1041	+	nonessential
**LigB**	Rv3062	3425584	3427107	1524	+	nonessential
**UvrD2**	Rv3198c	3569109	3571211	2103	-	essential
**AdnB**	Rv3201c	3573731	3577036	3306	-	essential
**AdnA**	Rv3202c	3577033	3580200	3168	-	nonessential
**Nei2**	Rv3297	3681320	3682087	768	+	nonessential
**DnaE2**	Rv3370c	3781501	3784740	3240	-	nonessential
**ImuB**	Rv3394c	3809442	3811025	1584	-	nonessential
**ImuA**	Rv3395c	3811022	3811636	615	-	nonessential
**RadA**	Rv3585	4026444	4027886	1443	+	nonessential
**MutY**	Rv3589	4030493	4031407	915	+	nonessential
**Nth**	Rv3674c	4115157	4115894	738	-	nonessential
**RecR**	Rv3715c	4159889	4160500	612	-	nonessential
**Prim-PolC**	Rv3730c	4180680	4181720	1041	-	nonessential
**LigC**	Rv3731	4181758	4182834	1077	+	nonessential
**MutT4**	Rv3908	4393449	4394195	747	+	nonessential

Built based on data from the Mycobrowser repository (https://mycobrowser.epfl.ch/)

Supplementary Table S3. Distribution of non-synonymous SNPs in genes related to DNA damage repair according to drug resistance and the presence/absence of T2DM in the host.

Gen / Loci	Host without T2DM		Host with T2DM		Total (n=399) n %	Nucleotide change
	Sensitive isolated (n = 123) n %	Drug resistant isolated (n = 101) n %	Sensitive isolated (n = 100) n %	Drug resistant isolated (n = 75) n %		
**RecF / Rv0003**						
3352	1(0.8)	0(0.0)	0(0.0)	0(0.0)	1(0.3)	73C>G
3439	2(1.6)	1(1.0)	1(1.0)	0(0.0)	4(1.0)	160G>A
3526	1(0.8)	0(0.0)	0(0.0)	0(0.0)	1(0.3)	247G>A
4013	1(0.8)	0(0.0)	0(0.0)	1(1.3)	2(0.5)	734C>T
4080	0(0.0)	2(2.0)	0(0.0)	1(1.3)	3(0.8)	801G>T
4350	0(0.0)	0(0.0)	1(1.0)	3(4.0)	4(1.0)	1071G>C
**SSBa / Rv0054**						
58694	0(0.0)	0(0.0)	0(0.0)	1(1.3)	1(0.3)	109C>T
**PolD2 / Rv0269c**						
323867	0(0.0)	1(1.0)	0(0.0)	0(0.0)	1(0.3)	665A>C
323967	1(0.8)	0(0.0)	0(0.0)	0(0.0)	1(0.3)	565G>A
324160	0(0.0)	0(0.0)	0(0.0)	1(1.3)	1(0.3)	372T>G
**MutT3 / Rv0413**						
500013	0(0.0)	0(0.0)	1(1.0)	0(0.0)	1(0.3)	301G>A
500019	0(0.0)	1(1.0)	0(0.0)	0(0.0)	1(0.3)	307G>T
500085	0(0.0)	0(0.0)	1(1.0)	0(0.0)	1(0.3)	373G>A
500136	1(0.8)	0(0.0)	0(0.0)	0(0.0)	1(0.3)	424T>G
500221	0(0.0)	0(0.0)	1(1.0)	0(0.0)	1(0.3)	509C>T
500235	0(0.0)	0(0.0)	1(1.0)	0(0.0)	1(0.3)	523C>T
**XthA / Rv0427c**						
516142	0(0.0)	0(0.0)	0(0.0)	1(1.3)	1(0.3)	751G>A
516408	1(0.8)	0(0.0)	0(0.0)	0(0.0)	1(0.3)	485C>T
516532	0(0.0)	0(0.0)	0(0.0)	1(1.3)	1(0.3)	361G>T
516655	2(1.6)	2(2.0)	0(0.0)	0(0.0)	4(1.0)	238G>C
516667	0(0.0)	0(0.0)	0(0.0)	1(1.3)	1(0.3)	226G>A
**RecD / Rv0629c**						
720502	1(0.8)	0(0.0)	0(0.0)	0(0.0)	1(0.3)	1231G>A
720981	0(0.0)	0(0.0)	0(0.0)	1(1.3)	1(0.3)	752C>T
721270	0(0.0)	0(0.0)	0(0.0)	1(1.3)	1(0.3)	463C>T
721311	0(0.0)	1(1.0)	0(0.0)	0(0.0)	1(0.3)	422A>C
721314	1(0.8)	0(0.0)	0(0.0)	0(0.0)	1(0.3)	419C>T
721476	1(0.8)	1(1.0)	0(0.0)	0(0.0)	2(0.5)	257A>C
721598	0(0.0)	0(0.0)	1(1.0)	1(1.3)	2(0.5)	135G>T
**RecB / Rv0630c**						
722205	1(0.8)	0(0.0)	0(0.0)	0(0.0)	1(0.3)	2809G>A
722304	0(0.0)	0(0.0)	0(0.0)	1(1.3)	1(0.3)	2710G>A
722984	2(1.6)	0(0.0)	0(0.0)	0(0.0)	2(0.5)	2030T>C
723202	1(0.8)	0(0.0)	0(0.0)	0(0.0)	1(0.3)	1812G>C
723492	1(0.8)	0(0.0)	0(0.0)	0(0.0)	1(0.3)	1522C>T

723828	2(1.6)	1(1.0)	1(1.0)	0(0.0)	4(1.0)	1186C>T
724307	3(2.4)	0(0.0)	0(0.0)	1(1.3)	4(1.0)	707G>A
724355	0(0.0)	0(0.0)	0(0.0)	1(1.3)	1(0.3)	659G>A
724487	1(0.8)	0(0.0)	0(0.0)	0(0.0)	1(0.3)	527C>T
**RecC / Rv0631c**						
725074	0(0.0)	0(0.0)	1(1.0)	0(0.0)	1(0.3)	3233G>A
725185	2(1.6)	0(0.0)	0(0.0)	0(0.0)	2(0.5)	3122C>T
725378	0(0.0)	2(2.0)	0(0.0)	0(0.0)	2(0.5)	2929G>T
725423	0(0.0)	0(0.0)	1(1.0)	0(0.0)	1(0.3)	2884T>C
725600	1(0.8)	0(0.0)	0(0.0)	0(0.0)	1(0.3)	2707G>C
725821	1(0.8)	0(0.0)	0(0.0)	0(0.0)	1(0.3)	2486A>C
725948	0(0.0)	0(0.0)	1(1.0)	0(0.0)	1(0.3)	2359G>A
726063	2(1.6)	0(0.0)	0(0.0)	0(0.0)	2(0.5)	2244G>C
726335	0(0.0)	0(0.0)	1(1.0)	0(0.0)	1(0.3)	1972A>G
726473	1(0.8)	0(0.0)	0(0.0)	0(0.0)	1(0.3)	1834G>A
726703*	32(26.0)	27(26.7)	32(32.0)	26(34.6)	117(29.3)	1604G>T
726816*	2(1.6)	5(4.9)	3(3.0)	10(13.3)	20(5.0)	1491C>G
727394	0(0.0)	0(0.0)	0(0.0)	1(1.3)	1(0.3)	913G>A
728162	0(0.0)	1(1.0)	0(0.0)	0(0.0)	1(0.3)	145C>T
**End (Nfo) / Rv0670**						
769859	0(0.0)	0(0.0)	1(1.0)	0(0.0)	1(0.3)	68T>C
770024	0(0.0)	1(1.0)	0(0.0)	0(0.0)	1(0.3)	233T>G
770087	0(0.0)	1(1.0)	0(0.0)	0(0.0)	1(0.3)	296A>G
770251	0(0.0)	0(0.0)	1(1.0)	0(0.0)	1(0.3)	460G>A
770277	0(0.0)	0(0.0)	0(0.0)	1(1.3)	1(0.3)	486C>A
770294	0(0.0)	0(0.0)	1(1.0)	0(0.0)	1(0.3)	503C>T
**Ku / Rv0937c**						
1045287	0(0.0)	0(0.0)	0(0.0)	1(1.3)	1(0.3)	734C>T
**LigD / Rv0938**						
1046255	1(0.8)	0(0.0)	0(0.0)	0(0.0)	1(0.3)	120G>A
1046362	0(0.0)	0(0.0)	1(1.0)	1(1.3)	2(0.5)	227T>G
1046506	1(0.8)	0(0.0)	0(0.0)	0(0.0)	1(0.3)	371G>A
1046716	1(0.8)	0(0.0)	0(0.0)	0(0.0)	1(0.3)	581T>C
1046736	0(0.0)	0(0.0)	1(1.0)	0(0.0)	1(0.3)	601C>T
1046757	2(1.6)	1(1.0)	6(6.0)	4(5.3)	13(3.3)	622C>T
1047039	0(0.0)	0(0.0)	1(1.0)	0(0.0)	1(0.3)	904C>A
1047087	0(0.0)	1(1.0)	0(0.0)	0(0.0)	1(0.3)	952C>T
1047165*	49(39.8)	25(24.8)	19(19.0)	11(14.7)	104(26.1)	1030C>T
1047283	0(0.0)	0(0.0)	1(1.0)	0(0.0)	1(0.3)	1148C>T
1047312	1(0.8)	0(0.0)	0(0.0)	0(0.0)	1(0.3)	1177A>G
1047393	0(0.0)	0(0.0)	1(1.0)	0(0.0)	1(0.3)	1258C>A
1047683*	0(0.0)	0(0.0)	4(4.0)	5(6.7)	9(2.3)	1548G>T
1047721	0(0.0)	0(0.0)	1(1.0)	0(0.0)	1(0.3)	1586G>C
**Fpg2 / Rv0944**						
1053955	0(0.0)	1(1.0)	0(0.0)	0(0.0)	1(0.3)	191C>T
1054197	2(1.6)	1(1.0)	0(0.0)	0(0.0)	3(0.8)	433G>A
**UvrD1 / Rv0949**						
1058642	1(0.8)	0(0.0)	0(0.0)	0(0.0)	1(0.3)	383A>G

1059280	0(0.0)	0(0.0)	1(1.0)	0(0.0)	1(0.3)	1021G>A
1059698	1(0.8)	0(0.0)	0(0.0)	0(0.0)	1(0.3)	1439C>G
1059736	0(0.0)	1(1.0)	0(0.0)	0(0.0)	1(0.3)	1477T>G
1059853	0(0.0)	0(0.0)	1(1.0)	0(0.0)	1(0.3)	1594C>G
1060121	0(0.0)	0(0.0)	1(1.0)	0(0.0)	1(0.3)	1862A>C
1060252	0(0.0)	1(1.0)	0(0.0)	0(0.0)	1(0.3)	1993C>T
1060473	1(0.8)	0(0.0)	0(0.0)	0(0.0)	1(0.3)	2214G>C
1060543	2(1.6)	0(0.0)	0(0.0)	0(0.0)	2(0.5)	2284A>C
1060571	0(0.0)	0(0.0)	1(1.0)	0(0.0)	1(0.3)	2312T>A
**Mfd / Rv1020**						
1139102*	0(0.0)	1(1.0)	1(1.0)	0(0.0)	2(0.5)	136G>A
1139327	0(0.0)	0(0.0)	0(0.0)	1(1.3)	1(0.3)	361C>T
1139498	0(0.0)	0(0.0)	0(0.0)	1(1.3)	1(0.3)	532C>G
1139840	1(0.8)	0(0.0)	0(0.0)	0(0.0)	1(0.3)	874G>A
1140026	0(0.0)	1(1.0)	0(0.0)	1(1.3)	2(0.5)	1060C>G
1140446	0(0.0)	0(0.0)	1(1.0)	0(0.0)	1(0.3)	1480G>A
1140610	0(0.0)	1(1.0)	0(0.0)	0(0.0)	1(0.3)	1644G>T
1140653	0(0.0)	0(0.0)	1(1.0)	1(1.3)	2(0.5)	1687G>C
1141107	0(0.0)	0(0.0)	1(1.0)	0(0.0)	1(0.3)	2141T>G
1141593	0(0.0)	1(1.0)	0(0.0)	0(0.0)	1(0.3)	2627G>T
1141649	0(0.0)	0(0.0)	1(1.0)	0(0.0)	1(0.3)	2683G>A
1141862	1(0.8)	0(0.0)	0(0.0)	0(0.0)	1(0.3)	2896C>T
1142671	0(0.0)	1(1.0)	0(0.0)	0(0.0)	1(0.3)	3705A>G
**MazG / Rv1021**						
1142725	0(0.0)	0(0.0)	0(0.0)	1(1.3)	1(0.3)	55A>G
1142939	0(0.0)	0(0.0)	1(1.0)	0(0.0)	1(0.3)	269C>T
1143599	0(0.0)	0(0.0)	1(1.0)	0(0.0)	1(0.3)	929C>A
**MutT2 / Rv1160**						
1286766	0(0.0)	0(0.0)	2(2.0)	0(0.0)	2(0.5)	172G>C
1286927	17(13.8)	0(0.0)	1(1.0)	0(0.0)	18(4.5)	333C>A
**TagA / Rv1210**						
1353660	0(0.0)	1(1.0)	0(0.0)	0(0.0)	1(0.3)	139C>A
1353726	0(0.0)	0(0.0)	1(1.0)	0(0.0)	1(0.3)	205G>C
1353850	1(0.8)	2(2.0)	0(0.0)	0(0.0)	3(0.8)	329G>A
1353951	0(0.0)	1(1.0)	0(0.0)	0(0.0)	1(0.3)	430T>C
1354050	0(0.0)	0(0.0)	1(1.0)	1(1.3)	2(0.5)	529G>C
**UdgB / Rv1259**						
1407489	0(0.0)	0(0.0)	0(0.0)	1(1.3)	1(0.3)	151C>T
1407519	0(0.0)	1(1.0)	0(0.0)	0(0.0)	1(0.3)	181G>A
1407532	1(0.8)	0(0.0)	0(0.0)	0(0.0)	1(0.3)	194C>T
1407604	0(0.0)	1(1.0)	0(0.0)	0(0.0)	1(0.3)	266G>A
1407993	0(0.0)	1(1.0)	0(0.0)	0(0.0)	1(0.3)	655T>G
**Ogt / adaB / Rv1316c**						
1477142	0(0.0)	1(1.0)	0(0.0)	0(0.0)	1(0.3)	490T>G
1477322	0(0.0)	0(0.0)	1(1.0)	0(0.0)	1(0.3)	310G>T
1477359	0(0.0)	1(1.0)	0(0.0)	0(0.0)	1(0.3)	273A>T
1477588*	24(19.5)	13(12.9)	29(29.0)	18(24.0)	84(21.1)	44C>G
**AlkA / Rv1317c**						

1477857	1(0.8)	0(0.0)	0(0.0)	0(0.0)	1(0.3)	1262G>A
1477870	0(0.0)	0(0.0)	1(1.0)	0(0.0)	1(0.3)	1249G>A
1478046	0(0.0)	1(1.0)	0(0.0)	0(0.0)	1(0.3)	1073C>T
1478182	1(0.8)	4(4.0)	4(4.0)	4(5.3)	13(3.3)	937C>T
1478274	0(0.0)	0(0.0)	1(1.0)	0(0.0)	1(0.3)	845T>C
1478435	2(1.6)	1(1.0)	0(0.0)	0(0.0)	3(0.8)	684A>C
1478851	0(0.0)	0(0.0)	1(1.0)	0(0.0)	1(0.3)	268A>C
1479085*	123(100.0)	101(100.0)	99(99.0)	75(100.0)	398(99.7)	34G>A
**NucS / Rv1321**						
1484393	0(0.0)	0(0.0)	0(0.0)	3(4.0)	3(0.8)	115A>G
1484430	1(0.8)	0(0.0)	0(0.0)	0(0.0)	1(0.3)	152A>G
1484439	0(0.0)	1(1.0)	0(0.0)	0(0.0)	1(0.3)	161G>T
**UvrC / Rv1420**						
1594115	0(0.0)	1(1.0)	0(0.0)	0(0.0)	1(0.3)	74A>G
1594453	0(0.0)	0(0.0)	0(0.0)	1(1.3)	1(0.3)	412T>C
1594736	0(0.0)	0(0.0)	0(0.0)	1(1.3)	1(0.3)	695G>A
1594906	0(0.0)	0(0.0)	2(2.0)	0(0.0)	2(0.5)	865G>A
1595067	0(0.0)	0(0.0)	1(1.0)	0(0.0)	1(0.3)	1026G>C
1595342	0(0.0)	0(0.0)	2(2.0)	0(0.0)	2(0.5)	1301T>C
1595357	0(0.0)	0(0.0)	1(1.0)	0(0.0)	1(0.3)	1316T>G
1595416	1(0.8)	0(0.0)	0(0.0)	0(0.0)	1(0.3)	1375T>G
1595887	0(0.0)	1(1.0)	0(0.0)	0(0.0)	1(0.3)	1846G>C
**DinB1 / Rv1537**						
1740274	0(0.0)	0(0.0)	0(0.0)	1(1.3)	1(0.3)	434C>T
1740399	0(0.0)	1(1.0)	0(0.0)	0(0.0)	1(0.3)	559A>G
1740504	2(1.6)	0(0.0)	0(0.0)	0(0.0)	2(0.5)	664A>C
1740694	1(0.8)	0(0.0)	0(0.0)	0(0.0)	1(0.3)	854C>T
1740771*	32(26.0)	27(26.7)	32(32.0)	26(34.7)	117(29.3)	931A>C
1740948	0(0.0)	0(0.0)	1(1.0)	0(0.0)	1(0.3)	1108C>T
**DnaE1 / Rv1547**						
1747846	0(0.0)	0(0.0)	2(2.0)	0(0.0)	2(0.5)	153G>A
1749516	0(0.0)	0(0.0)	1(1.0)	1(1.3)	2(0.5)	1823G>C
1749546	0(0.0)	0(0.0)	0(0.0)	3(4.0)	3(0.8)	1853C>G
1749684	0(0.0)	0(0.0)	1(1.0)	0(0.0)	1(0.3)	1991C>G
1750406	0(0.0)	0(0.0)	0(0.0)	1(1.3)	1(0.3)	2713A>G
1750484	1(0.8)	0(0.0)	0(0.0)	0(0.0)	1(0.3)	2791G>A
1750502	1(0.8)	1(1.0)	0(0.0)	0(0.0)	2(0.5)	2809G>C
1750602	2(1.6)	0(0.0)	0(0.0)	0(0.0)	2(0.5)	2909A>G
**PolA / Rv1629**						
1830915	3(2.4)	1(1.0)	1(1.0)	1(1.3)	6(1.5)	251A>G
1830918	1(0.8)	0(0.0)	0(0.0)	0(0.0)	1(0.3)	254C>T
1831242	0(0.0)	1(1.0)	0(0.0)	0(0.0)	1(0.3)	578T>G
1831298	1(0.8)	2(2.0)	0(0.0)	0(0.0)	3(0.8)	634G>A
1831340	25(20.3)	22(21.8)	3(3.0)	7(9.3)	57(14.3)	676C>T
1831445	0(0.0)	3(3.0)	0(0.0)	0(0.0)	3(0.8)	781C>T
1831730	2(1.60	2(2.0)	0(0.0)	0(0.0)	4(1.0)	1066A>G
1831978	2(1.6)	0(0.0)	0(0.0)	0(0.0)	2(0.5)	1314C>G
1832209	2(1.6)	0(0.0)	0(0.0)	0(0.0)	2(0.5)	1545G>A

1832777	0(0.0)	0(0.0)	1(1.0)	0(0.0)	1(0.3)	2113G>C
**UvrB / Rv1633**						
1837172	0(0.0)	0(0.0)	0(0.0)	1(1.3)	1(0.3)	98G>A
1837552	1(0.8)	0(0.0)	0(0.0)	0(0.0)	1(0.3)	478T>G
1837613	0(0.0)	2(2.0)	0(0.0)	7(9.3)	9(2.3)	539A>G
1837936	1(0.8)	0(0.0)	0(0.0)	0(0.0)	1(0.3)	862C>T
1838018	0(0.0)	0(0.0)	1(1.0)	0(0.0)	1(0.3)	944A>G
1838058	2(1.6)	0(0.0)	0(0.0)	0(0.0)	2(0.5)	984C>A
1838153*	0(0.0)	0(0.0)	5(5.0)	5(6.7)	10(2.5)	1079G>A
1838429	0(0.0)	0(0.0)	0(0.0)	1(1.3)	1(0.3)	1355T>C
1838672	1(0.8)	0(0.0)	0(0.0)	0(0.0)	1(0.3)	1598G>A
**UvrA / Rv1638**						
1844143	0(0.0)	14(13.9)	0(0.0)	5(6.7)	19(4.8)	403C>A
1844632	1(0.8)	1(1.0)	0(0.0)	1(1.3)	3(0.8)	892C>A
1845196	0(0.0)	0(0.0)	1(1.0)	0(0.0)	1(0.3)	1456G>A
1845524	3(2.4)	0(0.0)	1(1.0)	0(0.0)	4(1.0)	1784T>C
1846051	0(0.0)	0(0.0)	0(0.0)	1(1.3)	1(0.3)	2311G>A
1846157	0(0.0)	0(0.0)	3(3.0)	0(0.0)	3(0.8)	2417T>C
1846541	0(0.0)	0(0.0)	1(1.0)	0(0.0)	1(0.3)	2801C>T
**Mpg / Rv1688**						
1912998	0(0.0)	1(1.0)	0(0.0)	0(0.0)	1(0.3)	20C>T
1913172	0(0.0)	0(0.0)	0(0.0)	1(1.3)	1(0.3)	194G>C
**RecN / Rv1696**						
1920296	0(0.0)	1(1.0)	0(0.0)	0(0.0)	1(0.3)	614A>G
1920374	0(0.0)	0(0.0)	1(1.0)	0(0.0)	1(0.3)	692G>A
1920490	1(0.8)	0(0.0)	0(0.0)	0(0.0)	1(0.3)	808G>A
1920580	0(0.0)	0(0.0)	1(1.0)	0(0.0)	1(0.3)	898G>C
1921019	0(0.0)	0(0.0)	1(1.0)	0(0.0)	1(0.3)	1337G>A
1921099	0(0.0)	0(0.0)	1(1.0)	0(0.0)	1(0.3)	1417C>A
**Rv2119**						
2378813	0(0.0)	1(1.0)	3(3.0)	0(0.0)	4(1.0)	428A>G
2378938	1(0.8)	1(1.0)	0(0.0)	0(0.0)	2(0.5)	553G>A
**Cho / Rv2191**						
2453879	25(20.3)	22(21.8)	3(3.0)	7(9.3)	57(14.3)	61G>A
2453916	0(0.0)	0(0.0)	1(1.0)	0(0.0)	1(0.3)	98C>T
2454443	0(0.0)	0(0.0)	1(1.0)	0(0.0)	1(0.3)	625G>A
2454447	0(0.0)	0(0.0)	0(0.0)	1(1.3)	1(0.3)	629T>G
2454584	1(0.8)	0(0.0)	0(0.0)	0(0.0)	1(0.3)	766C>T
2454716	15(12.2)	0(0.0)	0(0.0)	0(0.0)	15(3.8)	898C>G
2455194	0(0.0)	0(0.0)	0(0.0)	1(1.3)	1(0.3)	1376G>A
**RNaseH1 / Rv2228c**						
2502051	1(0.8)	0(0.0)	0(0.0)	0(0.0)	1(0.3)	688G>A
2502416	0(0.0)	0(0.0)	1(1.0)	0(0.0)	1(0.3)	323G>T
2502446	3(2.4)	0(0.0)	0(0.0)	0(0.0)	3(0.8)	293A>G
**RecO / Rv2362c**						
2643642	0(0.0)	1(1.0)	0(0.0)	0(0.0)	1(0.3)	617C>T
2643677	0(0.0)	1(1.0)	0(0.0)	0(0.0)	1(0.3)	582C>A
2643915	6(4.9)	2(2.0)	0(0.0)	0(0.0)	8(2.0)	344A>T

2643954	0(0.0)	5(5.0)	0(0.0)	0(0.0)	5(1.3)	305G>A
2644083	1(0.8)	0(0.0)	0(0.0)	0(0.0)	1(0.3)	176T>C
2644239	0(0.0)	0(0.0)	1(1.0)	0(0.0)	1(0.3)	20G>A
**Nei1 / Rv2464c**						
2766900	0(0.0)	0(0.0)	2(2.0)	0(0.0)	2(0.5)	766G>A
2766941	0(0.0)	0(0.0)	1(1.0)	0(0.0)	1(0.3)	725C>T
2767200	1(0.8)	1(1.0)	0(0.0)	0(0.0)	2(0.5)	466A>G
2767206	2(1.6)	0(0.0)	0(0.0)	0(0.0)	2(0.5)	460C>T
2767338	1(0.8)	0(0.0)	0(0.0)	0(0.0)	1(0.3)	328G>A
2767631*	1(0.8)	1(1.0)	8(8.0)	0(0.0)	10(2.5)	35G>A
2767637	0(0.0)	0(0.0)	0(0.0)	1(1.3)	1(0.3)	29T>G
**SSBb / Rv2478c**						
2784248	0(0.0)	0(0.0)	0(0.0)	1(1.3)	1(0.3)	361C>G
2784541	1(0.8)	0(0.0)	0(0.0)	0(0.0)	1(0.3)	68G>A
**RuvB / Rv2592c**						
2923203	0(0.0)	0(0.0)	0(0.0)	2(2.7)	2(0.5)	1031A>G
2923395	0(0.0)	0(0.0)	2(2.0)	0(0.0)	2(0.5)	839G>T
2923832	0(0.0)	0(0.0)	0(0.0)	1(1.3)	1(0.3)	402C>G
2924143	1(0.8)	0(0.0)	0(0.0)	0(0.0)	1(0.3)	91G>A
2924202	0(0.0)	1(1.0)	0(0.0)	0(0.0)	1(0.3)	32C>T
2924212	0(0.0)	1(1.0)	6(6.0)	0(0.0)	7(1.8)	22G>A
**RuvA / Rv2593c**						
2924448	1(0.8)	0(0.0)	0(0.0)	0(0.0)	1(0.3)	373T>G
2924594	0(0.0)	1(1.0)	0(0.0)	0(0.0)	1(0.3)	227C>T
2924597	0(0.0)	0(0.0)	0(0.0)	1(1.3)	1(0.3)	224T>G
**RuvC / Rv2594c**						
2924968	0(0.0)	1(1.0)	0(0.0)	0(0.0)	1(0.3)	416C>T
2925271	0(0.0)	0(0.0)	1(1.0)	0(0.0)	1(0.3)	113C>T
2925296	0(0.0)	0(0.0)	1(1.0)	0(0.0)	1(0.3)	88G>A
**RecGwed / Rv2694c**						
3011490	0(0.0)	0(0.0)	1(1.0)	0(0.0)	1(0.3)	278C>T
3011653	0(0.0)	24(23.8)	0(0.0)	6(8.0)	30(7.5)	115A>G
3011692*	24(19.5)	13(12.9)	29(29.0)	18(24.0)	84(21.1)	76T>G
**Dut / Rv2697c**						
3013784*	24(19.5)	13(12.9)	29(29.0)	18(24.0)	84(21.1)	364G>C
**RecX / Rv2736c**						
3048735	0(0.0)	0(0.0)	2(2.0)	0(0.0)	2(0.5)	352A>G
3048773	0(0.0)	0(0.0)	0(0.0)	1(1.3)	1(0.3)	314G>A
**RecA / Rv2737c**						
3049792	0(0.0)	0(0.0)	1(1.0)	0(0.0)	1(0.3)	1633G>C
3050166	0(0.0)	1(1.0)	0(0.0)	0(0.0)	1(0.3)	1259C>T
3050289	0(0.0)	2(2.0)	0(0.0)	0(0.0)	2(0.5)	1136T>C
3050355	0(0.0)	0(0.0)	1(1.0)	0(0.0)	1(0.3)	1070C>T
3050400	0(0.0)	0(0.0)	1(1.0)	0(0.0)	1(0.3)	1025A>C
**RNaseH2 / Rv2902c**						
3212185	1(0.8)	0(0.0)	0(0.0)	0(0.0)	1(0.3)	772A>C
3212281	0(0.0)	0(0.0)	1(1.0)	0(0.0)	1(0.3)	676G>T
3212308	0(0.0)	0(0.0)	1(1.0)	0(0.0)	1(0.3)	649C>T

3212544	1(0.8)	0(0.0)	0(0.0)	0(0.0)	1(0.3)	413C>T
3212587	1(0.8)	0(0.0)	0(0.0)	0(0.0)	1(0.3)	370G>C
3212929	1(0.8)	0(0.0)	0(0.0)	0(0.0)	1(0.3)	28G>A
**MutM (Fpg) / Rv2924c**						
3238873	0(0.0)	0(0.0)	1(1.0)	0(0.0)	1(0.3)	598C>G
3238890	1(0.8)	0(0.0)	0(0.0)	0(0.0)	1(0.3)	581C>T
3239260	4(3.3)	0(0.0)	0(0.0)	0(0.0)	4(1.0)	211G>A
3239262	0(0.0)	0(0.0)	1(1.0)	4(5.3)	5(1.3)	209C>T
**RecG / Rv2973c**						
3328451	0(0.0)	0(0.0)	1(1.0)	0(0.0)	1(0.3)	1496C>T
3328825	0(0.0)	1(1.0)	0(0.0)	0(0.0)	1(0.3)	1122C>G
3328826	0(0.0)	0(0.0)	0(0.0)	2(2.7)	2(0.5)	1121A>G
3329106	1(0.8)	0(0.0)	0(0.0)	0(0.0)	1(0.3)	841C>A
3329181	0(0.0)	0(0.0)	5(5.0)	0(0.0)	5(1.3)	766G>T
3329403	0(0.0)	0(0.0)	1(1.0)	0(0.0)	1(0.3)	544A>C
3329643	1(0.8)	0(0.0)	0(0.0)	0(0.0)	1(0.3)	304G>T
3329763	0(0.0)	0(0.0)	1(1.0)	0(0.0)	1(0.3)	184A>C
**Ung / Rv2976c**						
3332221	0(0.0)	1(1.0)	0(0.0)	0(0.0)	1(0.3)	534G>A
3332634	1(0.8)	1(1.0)	0(0.0)	0(0.0)	2(0.5)	121A>G
**MutT1 / Rv2985**						
3342669	0(0.0)	0(0.0)	0(0.0)	1(1.3)	1(0.3)	505C>T
3342991	0(0.0)	1(1.0)	0(0.0)	0(0.0)	1(0.3)	827G>C
3343095	0(0.0)	0(0.0)	1(1.0)	0(0.0)	1(0.3)	931C>G
**LigA / Rv3014c**						
3372807	1(0.8)	0(0.0)	0(0.0)	0(0.0)	1(0.3)	1814A>G
3372835	0(0.0)	0(0.0)	1(1.0)	0(0.0)	1(0.3)	1786G>A
3372891	4(3.3)	0(0.0)	0(0.0)	0(0.0)	4(1.0)	1730T>C
3373152	2(1.6)	0(0.0)	0(0.0)	0(0.0)	2(0.5)	1469A>C
3373278	4(3.3)	0(0.0)	3(3.0)	0(0.0)	7(1.8)	1343G>T
3373329	0(0.0)	0(0.0)	0(0.0)	1(1.3)	1(0.3)	1292G>T
3373785	1(0.8)	0(0.0)	0(0.0)	0(0.0)	1(0.3)	836G>A
3374196	0(0.0)	1(1.0)	0(0.0)	0(0.0)	1(0.3)	425C>G
3374277	1(0.8)	2(2.0)	2(2.0)	4(5.3)	9(2.3)	344C>T
**DinB2 / Rv3056**						
3417147	0(0.0)	0(0.0)	1(1.0)	0(0.0)	1(0.3)	443T>G
3417668	1(0.8)	0(0.0)	0(0.0)	0(0.0)	1(0.3)	964G>A
**LigB / Rv3062**						
3425854	1(0.8)	0(0.0)	0(0.0)	1(1.3)	2(0.5)	271T>C
3425863	0(0.0)	0(0.0)	1(1.0)	1(1.3)	2(0.5)	280G>A
3425864	0(0.0)	0(0.0)	1(1.0)	0(0.0)	1(0.3)	281G>A
3425996	0(0.0)	0(0.0)	1(1.0)	0(0.0)	1(0.3)	413C>G
3426025*	2(1.6)	0(0.0)	2(2.0)	0(0.0)	4(1.0)	442G>A
3426157	0(0.0)	0(0.0)	3(3.0)	0(0.0)	3(0.8)	574G>A
**UvrD2 / Rv3198c**						
3569174	0(0.0)	0(0.0)	2(2.0)	0(0.0)	2(0.5)	2038A>G
3569723	0(0.0)	0(0.0)	0(0.0)	2(2.7)	2(0.5)	1489A>G
3569855	0(0.0)	1(1.0)	0(0.0)	0(0.0)	1(0.3)	1357G>A

3569950	1(0.8)	0(0.0)	0(0.0)	0(0.0)	1(0.3)	1262C>G
3571142	0(0.0)	1(1.0)	0(0.0)	0(0.0)	1(0.3)	70C>T
**AdnB / Rv3201c**						
3574504*	13(10.6)	31(30.7)	0(0.0)	7(9.3)	51(12.8)	2533C>T
3574569	1(0.8)	0(0.0)	0(0.0)	0(0.0)	1(0.3)	2468G>T
3575077	0(0.0)	0(0.0)	1(1.0)	0(0.0)	1(0.3)	1960A>T
3575106*	1(0.8)	1(1.0)	10(10.0)	0(0.0)	12(3.0)	1931T>C
3575476	0(0.0)	1(1.0)	0(0.0)	0(0.0)	1(0.3)	1561G>A
3575496	1(0.8)	0(0.0)	0(0.0)	0(0.0)	1(0.3)	1541C>T
3576231	0(0.0)	0(0.0)	2(2.0)	0(0.0)	2(0.5)	806A>G
3576568	0(0.0)	0(0.0)	0(0.0)	1(1.3)	1(0.3)	469G>A
3576667	0(0.0)	0(0.0)	1(1.0)	0(0.0)	1(0.3)	370C>T
3576865	0(0.0)	0(0.0)	1(1.0)	0(0.0)	1(0.3)	172G>C
3576877	1(0.8)	0(0.0)	0(0.0)	0(0.0)	1(0.3)	160G>A
**AdnA / Rv3202c**						
3577476	0(0.0)	0(0.0)	0(0.0)	1(1.3)	1(0.3)	2725A>G
3577958*	123(100.0)	100(99.0)	99(99.0)	75(100.0)	397(99.5)	2243C>T
3578516	1(0.8)	0(0.0)	0(0.0)	0(0.0)	1(0.3)	1685G>T
3579416	0(0.0)	0(0.0)	0(0.0)	1(1.3)	1(0.3)	785C>G
**Nei2 / Rv3297**						
3681377	1(0.8)	0(0.0)	0(0.0)	0(0.0)	1(0.3)	58G>A
3681543	0(0.0)	0(0.0)	0(0.0)	1(1.3)	1(0.3)	224G>A
3681715	1(0.8)	0(0.0)	0(0.0)	0(0.0)	1(0.3)	396G>A
3682014	1(0.8)	0(0.0)	0(0.0)	0(0.0)	1(0.3)	695G>T
3682023	25(20.3)	22(21.8)	3(3.0)	7(9.3)	57(14.3)	704C>T
3682041	0(0.0)	0(0.0)	1(1.0)	0(0.0)	1(0.3)	722C>T
**DnaE2 / Rv3370c**						
3781574*	0(0.0)	0(0.0)	3(3.0)	0(0.0)	3(0.8)	3203C>G
3781575	0(0.0)	0(0.0)	0(0.0)	1(1.3)	1(0.3)	3202G>A
3781803	0(0.0)	0(0.0)	0(0.0)	1(1.3)	1(0.3)	2974G>A
3782087*	0(0.0)	0(0.0)	1(1.0)	0(0.0)	1(0.3)	2690C>T
3782994	0(0.0)	0(0.0)	0(0.0)	1(1.3)	1(0.3)	1783C>A
3783078	0(0.0)	1(1.0)	0(0.0)	0(0.0)	1(0.3)	1699G>A
3783114	0(0.0)	1(1.0)	0(0.0)	0(0.0)	1(0.3)	1663G>A
3783141	1(0.8)	0(0.0)	2(2.0)	3(4.0)	6(1.5)	1636A>C
3783663	0(0.0)	0(0.0)	1(1.0)	0(0.0)	1(0.3)	1114G>A
3783891	1(0.8)	0(0.0)	0(0.0)	0(0.0)	1(0.3)	886G>A
3784008	0(0.0)	0(0.0)	1(1.0)	0(0.0)	1(0.3)	769A>C
3784011	1(0.8)	1(1.0)	7(7.0)	0(0.0)	9(2.3)	766G>A
3784205	12(9.8)	14(13.9)	5(5.0)	3(4.0)	34(8.5)	572G>A
3784238	0(0.0)	1(1.0)	0(0.0)	0(0.0)	1(0.3)	539C>G
3784268	0(0.0)	0(0.0)	1(1.0)	0(0.0)	1(0.3)	509C>T
3784559	0(0.0)	2(2.0)	0(0.0)	8(10.7)	10(2.5)	218C>T
3784601	0(0.0)	0(0.0)	1(1.0)	0(0.0)	1(0.3)	176C>T
3784770	0(0.0)	1(1.0)	1(1.0)	0(0.0)	2(0.5)	7T>G
**ImuB / Rv3394c**						
3809500	0(0.0)	0(0.0)	1(1.0)	0(0.0)	1(0.3)	1526C>T
3809671	0(0.0)	0(0.0)	1(1.0)	0(0.0)	1(0.3)	1355C>T

3809929	0(0.0)	0(0.0)	1(1.0)	0(0.0)	1(0.3)	1097G>A
3809932	1(0.8)	0(0.0)	0(0.0)	0(0.0)	1(0.3)	1094A>C
3809953*	0(0.0)	0(0.0)	1(1.0)	0(0.0)	1(0.3)	1073G>T
**ImuA / Rv3395c**						
3811327	1(0.8)	0(0.0)	4(4.0)	2(2.7)	7(1.8)	310G>A
3811629*	123(100.0)	100(99.0)	99(99.0)	75(100.0)	397(99.5)	8T>C
**RadA / Rv3585**						
4026696	0(0.0)	0(0.0)	1(1.0)	0(0.0)	1(0.3)	253A>G
4026865	0(0.0)	0(0.0)	1(1.0)	0(0.0)	1(0.3)	422G>C
4027101	0(0.0)	2(2.0)	0(0.0)	0(0.0)	2(0.5)	658G>C
4027363	1(0.8)	0(0.0)	0(0.0)	0(0.0)	1(0.3)	920T>C
4027751	1(0.8)	0(0.0)	0(0.0)	0(0.0)	1(0.3)	1308G>A
**MutY / Rv3589**						
4030686	0(0.0)	1(1.0)	0(0.0)	1(1.3)	2(0.5)	194A>C
4030782	0(0.0)	0(0.0)	1(1.0)	0(0.0)	1(0.3)	290C>T
4030797	0(0.0)	0(0.0)	1(1.0)	0(0.0)	1(0.3)	305A>G
4030886	0(0.0)	1(1.0)	0(0.0)	0(0.0)	1(0.3)	394A>G
4030995	0(0.0)	1(1.0)	0(0.0)	0(0.0)	1(0.3)	503C>T
4031277	0(0.0)	14(13.9)	0(0.0)	5(6.7)	19(4.8)	785G>A
**Nth / Rv3674c**						
4115602	0(0.0)	1(1.0)	0(0.0)	0(0.0)	1(0.3)	293C>T
4115695	1(0.8)	0(0.0)	0(0.0)	0(0.0)	1(0.3)	200C>T
4115890	0(0.0)	0(0.0)	2(2.0)	0(0.0)	2(0.5)	5C>G
**RecR / Rv3715c**						
4160371	0(0.0)	0(0.0)	2(2.0)	0(0.0)	2(0.5)	130G>T
Prim-PolC	Rv3730c					
4180927	0(0.0)	0(0.0)	3(3.0)	0(0.0)	3(0.8)	794G>A
**LigC / Rv3731**						
4181974	0(0.0)	1(1.0)	1(1.0)	1(1.3)	3(0.8)	217T>G
4182348	0(0.0)	0(0.0)	1(1.0)	0(0.0)	1(0.3)	591G>A
4182407	1(0.8)	0(0.0)	0(0.0)	0(0.0)	1(0.3)	650A>G
4182512	0(0.0)	0(0.0)	0(0.0)	1(1.3)	1(0.3)	755G>A
4182521	0(0.0)	1(1.0)	0(0.0)	0(0.0)	1(0.3)	764T>C
4182695*	49(39.8)	24(23.8)	19(19.0)	11(14.7)	103(25.8)	938A>G
**MutT4 / Rv3908**						
4393666	0(0.0)	1(1.0)	0(0.0)	0(0.0)	1(0.3)	218A>G
4394097	0(0.0)	0(0.0)	1(1.0)	0(0.0)	1(0.3)	649C>G
4394181	1(0.8)	0(0.0)	0(0.0)	0(0.0)	1(0.3)	733G>T

*SNPs related to sublinage

Supplementary Table S4. Distribution of genes with SNPs according to drug resistance and absence/presence of T2DM in the host (continuation)

Gen	Host without T2DM		Total without T2DM (n = 224) n (%)	Host with T2DM		Total with T2DM (n = 175) n (%)	Total Sensitive isolate (n = 223) n (%)	Total Drug resistant isolate (n = 176) n (%)	Total (n = 399) n (%)	Exclusive sub-linage diversification
	Sensitive isolate (n = 123) n (%)	Drug resistant isolate (n = 101) n (%)		Sensitive isolate (n = 100) n (%)	Drug resistant isolate (n = 75) n (%)					
RecF	5(4.1)	3(3.0)	8(3.6)	2(2.0)	5(6.7)	7(4.0)	7(3.1)	8(4.5)	15(3.8)	No
SSBa	0(0.0)	0(0.0)	0(0.0)	0(0.0)	1(1.3)	1(0.6)	0(0.0)	1(0.6)	1(0.3)	4.1.2
PolD2	1(0.8)	1(1.0)	2(0.9)	0(0.0)	1(1.3)	1(0.6)	1(0.4)	2(1.1)	3(0.8)	No
MutT3	1(0.8)	1(1.0)	2(0.9)	4(4.0)	0(0.0)	4(2.3)	5(2.2)	1(0.6)	6(1.5)	No
XthA	3(2.4)	2(2.0)	5(2.2)	0(0.0)	3(4.0)	3(1.7)	3(1.3)	5(2.8)	8(2.0)	No
RecD	3(2.4)	2(2.0)	5(2.2)	1(1.0)	3(4.0)	4(2.3)	4(1.8)	5(2.8)	9(2.3)	No
RecB	11(8.9)	1(1.0)	12(5.4)	1(1.0)	3(4.0)	4(2.3)	12(5.4)	4(2.3)	16(4.0)	No
RecC•	7(5.7)	3(3.0)	10(4.5)	4(4.0)	1(1.3)	5(2.9)	11(4.9)	4(2.3)	15(3.8)	No
End (Nfo)	0(0.0)	2(2.0)	2(0.9)	3(3.0)	1(1.3)	4(2.3)	3(1.3)	3(1.7)	6(1.5)	No
Ku	0(0.0)	0(0.0)	0(0.0)	0(0.0)	1(1.3)	1(0.6)	0(0.0)	1(0.6)	1(0.3)	4.3.4.2
Fpg2	2(1.6)	2(2.0)	4(1.8)	0(0.0)	0(0.0)	0(0.0)	2(0.9)	2(1.1)	4(1.0)	No
UvrD1	5(4.1)	2(2.0)	7(3.1)	4(4.0)	0(0.0)	4(2.3)	9(4.0)	2(1.1)	11(2.8)	No
TagA	1(0.8)	4(4.0)	5(2.2)	2(2.0)	1(1.3)	3(1.7)	3(1.3)	5(2.8)	8(2.0)	No
UdgB	1(0.8)	3(3.0)	4(1.8)	0(0.0)	1(1.3)	1(0.6)	1(0.4)	4(2.3)	5(1.3)	No
Ogt / adaB•	0(0.0)	2(2.0)	2(0.9)	1(1.0)	0(0.0)	1(0.6)	1(0.4)	2(1.1)	3(0.8)	No
AlkA•	4(3.3)	6(5.9)	10(4.5)	7(7.0)	4(5.3)	11(6.3)	11(4.9)	10(5.7)	21(5.3)	No
NucS	1(0.8)	1(1.0)	2(0.9)	0(0.0)	3(4.0)	3(1.7)	1(0.4)	4(2.3)	5(1.3)	No
UvrC	1(0.8)	2(2.0)	3(1.3)	4(4.0)	2(2.7)	6(3.4)	5(2.2)	4(2.3)	9(2.3)	No
DinB1•	3(2.4)	1(1.0)	4(1.8)	1(1.0)	1(1.3)	2(1.1)	4(1.8)	2(1.1)	6(1.5)	No
UvrB•	5(4.1)	2(2.0)	7(3.1)	1(1.0)	9(12.0)	10(5.7)	6(2.7)	11(6.3)	17(4.3)	No
Mpg	0(0.0)	1(1.0)	1(0.4)	0(0.0)	1(1.3)	1(0.6)	0(0.0)	2(1.1)	2(0.5)	4.3.4.2 & 4.3.4.1
RecN	1(0.8)	1(1.0)	2(0.9)	3(3.0)	0(0.0)	3(1.7)	4(1.8)	1(0.6)	5(1.3)	No
Rv2119	1(0.8)	2(2.0)	3(1.3)	3(3.0)	0(0.0)	3(1.7)	4(1.8)	2(1.1)	6(1.5)	No
Nei1•	4(3.3)	1(1.0)	5(2.2)	3(3.0)	1(1.3)	4(2.3)	7(3.1)	2(1.1)	9(2.3)	No
SSBb	1(0.8)	0(0.0)	1(0.4)	0(0.0)	1(1.3)	1(0.6)	1(0.4)	1(0.6)	2(0.5)	No
RuvA	1(0.8)	1(1.0)	2(0.9)	0(0.0)	1(1.3)	1(0.6)	1(0.4)	2(1.1)	3(0.8)	No
RuvC	0(0.0)	1(1.0)	1(0.4)	2(2.0)	0(0.0)	2(1.1)	2(0.9)	1(0.6)	3(0.8)	No
Dut•	0(0.0)	0(0.0)	0(0.0)	0(0.0)	0(0.0)	0(0.0)	0(0.0)	0(0.0)	0(0.0)	No
RecA	0(0.0)	3(3.0)	3(1.3)	3(3.0)	0(0.0)	3(1.7)	3(1.3)	3(1.7)	6(1.5)	No
MutM (Fpg)	5(4.1)	0(0.0)	5(2.2)	2(2.0)	4(5.3)	6(3.4)	7(3.1)	4(2.3)	11(2.8)	No
Ung	1(0.8)	2(2.0)	3(1.3)	0(0.0)	0(0.0)	0(0.0)	1(0.4)	2(1.1)	3(0.8)	No
MutT1	0(0.0)	1(1.0)	1(0.4)	1(1.0)	1(1.3)	2(1.1)	1(0.4)	2(1.1)	3(0.8)	4.1.2 & 4.1.2.1
LigA	9(7.3)	3(3.0)	12(5.4)	6(6.0)	5(6.7)	11(6.3)	15(6.7)	8(4.5)	23(5.8)	No
DinB2	1(0.8)	0(0.0)	1(0.4)	1(1.0)	0(0.0)	1(0.6)	2(0.9)	0(0.0)	2(0.5)	4.3.2 & 4.3.3
UvrD2	1(0.8)	2(2.0)	3(1.3)	2(2.0)	2(2.7)	4(2.3)	3(1.3)	4(2.3)	7(1.8)	No
AdnB•	3(2.4)	1(1.0)	4(1.8)	4(4.0)	1(1.3)	5(2.9)	7(3.1)	2(1.1)	9(2.3)	No
AdnA•	1(0.8)	0(0.0)	1(0.4)	0(0.0)	2(2.7)	2(1.1)	1(0.4)	2(1.1)	3(0.8)	No
RadA	2(1.6)	2(2.0)	4(1.8)	2(2.0)	0(0.0)	2(1.1)	4(1.8)	2(1.1)	6(1.5)	No
Nth	1(0.8)	1(1.0)	2(0.9)	2(2.0)	0(0.0)	2(1.1)	3(1.3)	1(0.6)	4(1.0)	No
LigC•	2(1.6)	2(2.0)	4(1.8)	2(2.0)	2(2.7)	4(2.3)	4(1.8)	4(2.3)	8(2.0)	No
MutT4	1(0.8)	1(1.0)	2(0.9)	1(1.0)	0(0.0)	1(0.6)	2(0.9)	1(0.6)	3(0.8)	No

T2DM: Type 2 diabetes mellitus. • Lineage-related SNPs and SNPs >99% of the sample are excluded.
